# The Hexadehydro-Diels–Alder Reaction: A New Chapter in Aryne Chemistry

**DOI:** 10.1002/anie.201402405

**Published:** 2014-05-05

**Authors:** Catherine Holden (née Hall), Michael F Greaney

**Affiliations:** School of Chemistry, University of Manchester Oxford Rd, Manchester, M13 9PL (UK)

**Keywords:** arynes, cycloaddition, Diels–Alder reactions, reactive intermediates

**Scheme 1:**
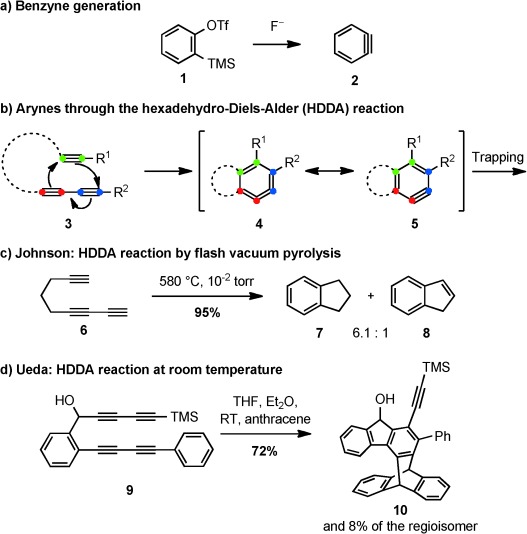
Generation of *ortho*-aryne intermediates from the established triflate precursor **1** (a) and through the hexadehydro-Diels–Alder (HDDA) reaction (b–d). OTf=trifluoromethanesulfonate, TMS=trimethylsilyl.

Arynes continue to stimulate new reactions and theoretical insight in aromatic chemistry. This has been driven in recent years by the exceptional versatility of 2-(trimethylsilyl)phenyl triflate (**1**) as a precursor to *ortho*-benzyne.[1] Such compounds react under mild conditions and have enabled a substantial development of aryne chemistry both in terms of classical transformations (nucleophilic addition, pericyclic reactions) and in opening up new areas, such as σ*-*insertion reactions and transition-metal-catalyzed processes. As with all methods for the generation of *ortho*-benzyne to date, compound **1** requires the removal of two adjacent groups from a pre-existing arene. Recent work from the groups of Hoye and Lee has utilized a completely different approach to *ortho*-arynes, which proceeds through the intramolecular cycloaddition of triynes **3** (Scheme 1).[2, 3] This transformation, which was termed a hexadehydro-Diels–Alder (HDDA) reaction by Hoye in analogy to other dehydropericyclic reactions, is perhaps surprising at first glance because of the infrequently depicted aryne resonance structure **4**, which immediately arises from the [4+2] cycloaddition. The trapping of an *ortho-*benzyne intermediate generated in this fashion can then be accomplished by a variety of inter- and intramolecular transformations, affording new approaches to valuable benzenoid compounds.

The origin of the HDDA reaction lies in two independent reports from the groups of Johnson and Ueda in 1997.[4] Johnson and co-workers subjected the simple hydrocarbon 1,3,8-nonatriyne (**6**) to flash vacuum pyrolysis and were able to isolate indane and indene in a combined yield of 95 % (Scheme 1 c).[4a] The most likely mechanism appeared to be a [4+2] cycloaddition to form an aryne, followed by reduction under the pyrolytic conditions. Deuterium labeling studies support this mechanism, ruling out a possible 1,2-shift/Bergman cyclization pathway. DFT calculations for the cycloaddition of acetylene with butadiyne gave the surprising result that the formation of benzyne through an HDDA reaction would be highly exothermic (−52 kcal mol^−1^) and accompanied by a large free energy of activation (37 kcal mol^−1^).[4c]

The Ueda group observed that tetraynes such as **9** could cyclize to 5*H*-fluorenol derivatives at room temperature, with the intermediacy of an *ortho*-benzyne species being established by intra- and intermolecular trapping experiments (to give **10**, for example).[4b] Ueda and co-workers then continued to investigate the scope of the HDDA reaction and established intramolecular trapping reactions with oxygen, sulfur, and nitrogen nucleophiles. Applications were demonstrated in the areas of helical polyene synthesis and in studies on the DNA-cleaving properties of the *ortho*-aryne generated in this fashion.[5] The similarities to the Bergman and Myers–Saito cycloaromatization reactions are evident, and cleavage of the DNA strands, analogous to the en–diyne *para*-benzyne processes, was observed by the Ueda group using simple oxidative triggering of the HDDA reaction.[5a,d]

**Scheme 2:**
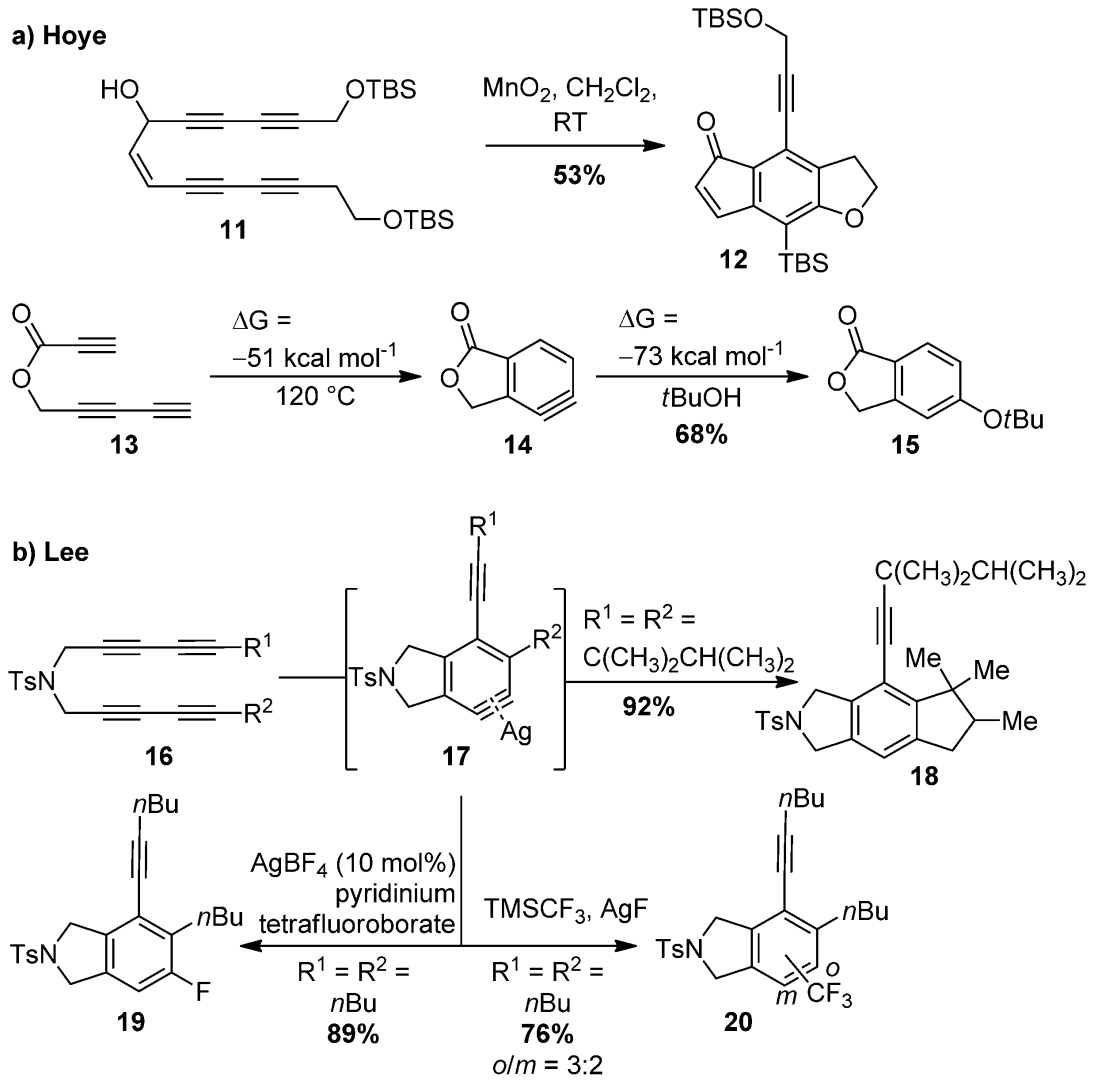
HDDA cycloaromatization reactions, described by the groups of Hoye and Lee. TBS=*tert*-butyldimethylsilyl, Ts=*para*-toluenesulfonyl.

Despite these impressive antecedents,[6] the potential of the HDDA approach to *ortho*-benzyne motifs was not widely appreciated until Hoye and co-workers published substantial extensions of this method in 2012.[2] They unexpectedly observed cycloaromatization of the oxidation product of tetrayne **11**, which afforded benzenoid **12** through *ortho*-aryne formation and trapping through a retro-Brook rearrangement of the silyl ether (Scheme 2 a). The substrate scope of this transformation is remarkably broad, as the reagent-free conditions were found to be suitable for a range of base-sensitive functional groups that would challenge conventional methods for aryne formation (e.g., silyl groups, which are problematic for reagent **1**, and esters, which are incompatible with metal–halogen exchange). Activation of the diynophile by electron-withdrawing groups was established, and ring-size effects and a wide variety of inter- and intramolecular trapping steps were studied. Successful HDDA and Diels–Alder trapping reactions were observed using CHCl_3_ as a solvent, which is an excellent hydrogen atom donor, pointing to a lack of radical character in the cyclization. DFT calculations for the HDDA cyclisation of **13** revealed a reaction profile that was quantitatively similar to that calculated by Johnson in earlier work and predicted the formation of the aryne intermediate to be exothermic by a substantial 51 kcal mol^−1^.

Following this report, Lee and co-workers discovered an HDDA system based on the silver-catalyzed cycloaromatization of tetraynes **16**.[3] The aryne-trapping event was a striking C(sp^3^)–H activation to form polycyclic products such as **18**; the silver catalyst is thought to form a metal-stabilized aryne **17** (an “isoindolyne”),[7] which is capable of alkane C–H activation (Scheme 2 b). The reaction was exemplified on a variety of secondary alkane C–H bonds and poly-yne structures; *gem*-dimethyl substitution on the developing carbocycle **18** was observed to facilitate the reaction. Mechanistic investigations pointed towards a concerted addition of the C–H bond across the aryne intermediate, a reaction with little precedent in carbocyclizations. For unsymmetric tetraynes, where regioisomeric arenes may be formed, moving the tethering NTs group to create a diynamide effectively activated this moiety to undergo an HDDA reaction.

**Scheme 3:**
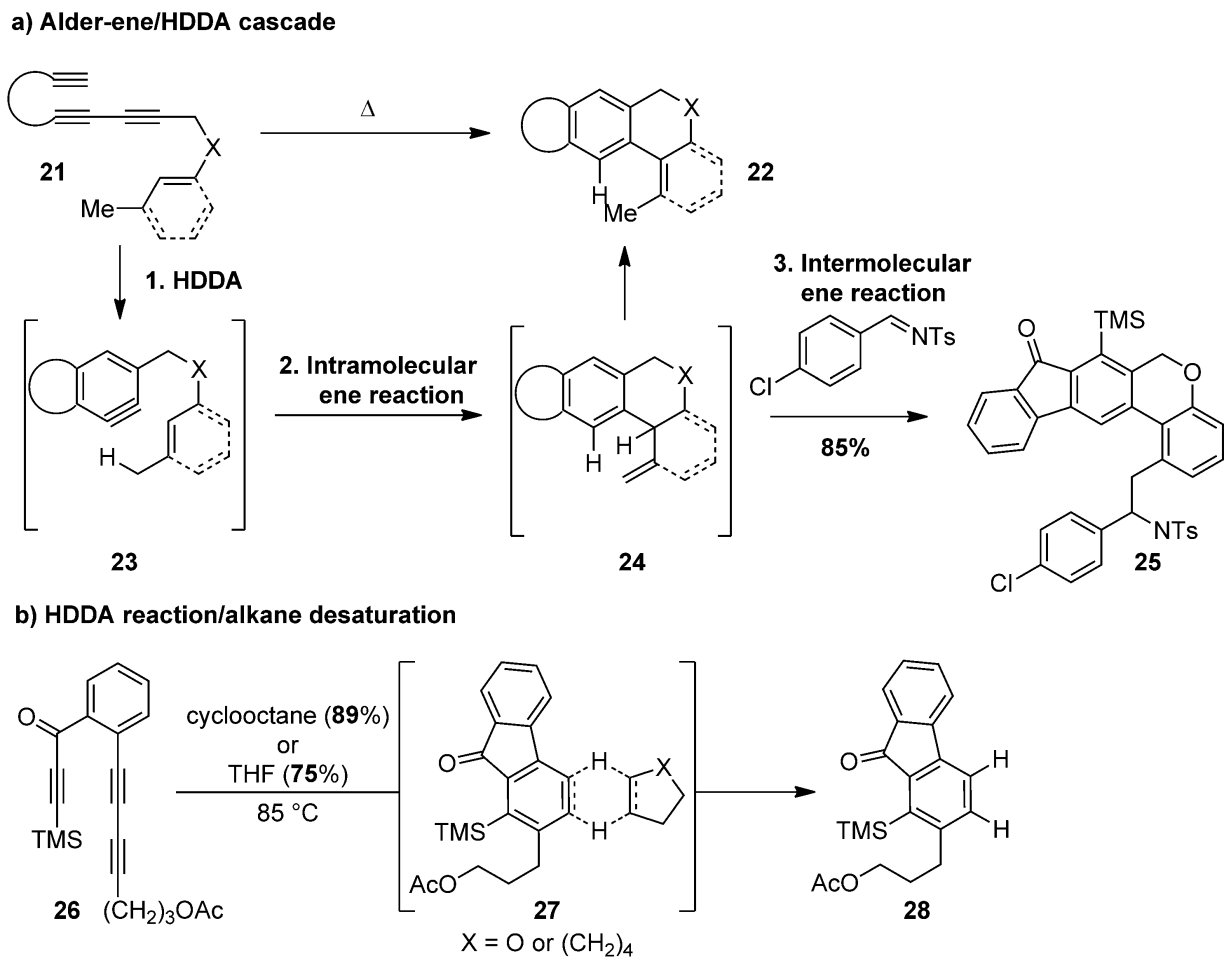
Alder–ene and alkane-desaturation trapping processes in the HDDA reaction.

Since these initial reports, the Hoye and Lee groups have rapidly developed a host of reaction motifs that showcase both the unique reactivity of arynes and the potential of the HDDA reaction in arene synthesis.[8, 9] Lee et al. have demonstrated that AgF, AgCF_3_, and AgSCF_3_ can all mediate HDDA reactions with incorporation of the respective fluorine-containing counterions to yield motifs that are valued in medicinal chemistry and agrochemistry.[9b] Such intermolecular trapping processes raise the prospect of regioisomeric products, a long-standing challenge in aryne addition chemistry. Excellent regioselectivities were observed for the fluorination reaction (fluorine incorporated *ortho* to the R^2^ group; Scheme 2 b); however, the addition of the SCF_3_ and CF_3_ groups tended to produce mixtures. The factors that control intermolecular trapping in the HDDA reaction have been the focus of a recent study by the Lee group, who have delineated the electronic and steric control elements that are in operation for a range of nitrogen- and oxygen-based nucleophiles.[9c] Furthermore, both the Hoye and Lee groups have demonstrated Alder–ene chemistry as a trapping event, with the Hoye group achieving impressive HDDA/aromatic ene/Alder–ene cascade sequences (Scheme 3 a).[8b, 9a] The use of an external enophile enabled the capture of the product of the first ene reaction, isotoluene **24**, which consecutively generated three rings in the absence of any external reagents and without the formation of any by-products (product **25**).

The Hoye group have reported a particularly eye-catching example of HDDA chemistry, which involves alkane desaturation (Scheme 3 b).[8a] Alkane desaturation is known to occur during a number of biological processes, but there is no general synthetic equivalent. In the presence of a range of cycloalkanes, an HDDA reaction led to the production of cycloalkenes through a concerted process; excellent yields were obtained for cyclooctane, -heptane, and -pentane donors. Interestingly, cyclohexane was a poor substrate for this reaction. DFT calculations suggest that the near-planar, concerted transition state of the reaction favors 2 H donors that have a higher degree of eclipsed low-energy conformations. Tetrahydrofuran (THF) was also shown to be an effective donor, which is a significant result in the wider context of aryne chemistry, as this solvent is frequently used with other *ortho*-benzyne precursors such as **1**. It is noteworthy that the HDDA cyclization enabled mechanistic insight into aryne reactivity that might be difficult to achieve using conventional precursors, where aryne generation is often rate-limiting, and additional reagents can obscure the analysis of subsequent steps.

In summary, the HDDA reaction offers a unique approach to *ortho*-arynes; all atoms of the starting triyne are conserved in the product, and diverse functional groups may be installed by strategic design of the trapping steps. These transformations offer exciting potential for the synthesis of complex benzenoids of the type found in natural products and functional materials, with some exceptional examples of atom-economic cascade processes having already been demonstrated. Allied with new mechanistic insights into the trapping steps, these reactions open up significant new areas of study and application for the continually fascinating field of aryne chemistry.
